# Emergent initiation of dialysis is related to an increase in both mortality and medical costs

**DOI:** 10.1038/s41598-020-76765-0

**Published:** 2020-11-12

**Authors:** Yuki Shimizu, Junichiro Nakata, Naotake Yanagisawa, Yuka Shirotani, Haruna Fukuzaki, Nao Nohara, Yusuke Suzuki

**Affiliations:** 1 Department of Nephrology, Juntendo University Faculty of Medicine, 2-1-1 Hongo, Bunkyo-ku, Tokyo, 113-8421 Japan; 2 Medical Technology Innovation Center, Juntendo University, Tokyo, Japan

**Keywords:** Health care, Medical research, Nephrology

## Abstract

The number of patients with end-stage renal disease (ESRD) has been increasing, with dialysis treatment being a serious economic problem. To date, no report in Japan considered medical costs spent at the initiation of dialysis treatment, although some reports in other countries described high medical costs in the first year. This study focused on patient status at the time of initiation of dialysis and examined how it affects prognosis and the medical costs. As a result, all patients dying within 4 months experienced emergent dialysis initiation. Emergent dialysis initiation and high medical costs were risk factors for death within 2 years. High C-reactive protein levels and emergent dialysis initiation were associated with increasing medical costs. Acute kidney injury (AKI) contributed most to emergent dialysis initiation followed by stroke, diabetes, heart failure, and short-term care by nephrologists. Therefore, emergent dialysis initiation was a contributing factor to both death and increasing medical costs. To avoid the requirement for emergent dialysis initiation, patients with ESRD should be referred to nephrologists earlier. Furthermore, ESRD patients with clinical histories of AKI, stroke, diabetes, or heart failure should be observed carefully and provided pre-planned initiation of dialysis.

## Introduction

The number of patients with end-stage renal disease (ESRD) has been increasing year by year worldwide^1–6^. Karopadi reported that in 2008, there were 1.75 million patients who regularly received renal replacement therapy (RRT) worldwide, of whom, 89% were on hemodialysis (HD) and 11% were on peritoneal dialysis (PD)^7^.

With the increase in the number of ESRD patients, medical costs for the treatment of chronic kidney disease (CKD) are rising. The costs are suggested to represent a leading threat to health care resources worldwide^7^. Peeters et al. described CKD and dialysis as not only medical problems, but also economic challenges. RRT consumes a lot of resources as the equipment and materials involved are expensive^8^. Therefore, across the globe, various healthcare reimbursement schemes have been implemented to improve dialysis care, while curtailing rising treatment expenditure^9^. For instance, reports from Hong Kong compared the RRT modalities and showed that hospital HD had the highest medical costs in the first year of initiation of dialysis treatment^10^. Indeed in Hong Kong, since 1995, the government has removed the upper age of limit of 55 years for subsidizing home dialysis consumables and continued the “PD First” policy^11^ and they seemed to achieve the expected result, i.e., increase in the percentage of patients utilizing PD. However, insufficient data are available to determine whether any of the approaches provide better quality of life or patient outcomes^12^ around the world, including in Japan.

To discuss the aforementioned medical and economic issues, it is necessary to consider the various economic conditions and health care systems in different countries. For example, Japanese universal health care system is characterized by compulsory affiliation, free access, low copayments, and coverage of costs by insurance premiums and public subsidies^13^. Moreover, in the Global Burden of Diseases, Injuries, and Risk Factors Study (GBD), Japan recorded the smallest range of subnational Healthcare Access and Quality performance in 2016^14^. This means that there is little domestic disparity in terms of access and quality of healthcare in the country. The elderly and physically handicapped, including dialysis patients, are further funded by copayments^15^. Each patient with ESRD makes a copayment of up to 10–20 thousand Japanese Yen (JPY) per month for full access to dialysis including all medications and consumables^16^. According to the latest Japanese Society for Dialysis Therapy (JSDT) Renal Data Registry^17^, there are more than 330,000 dialysis patients in Japan. Because of the costs involved in dialysis treatment (about 5 million JPY/patient/year^18^) and the national cost of medical care (43 trillion JPY/year) announced by the Ministry of Health, Labor, and Welfare, 4% of the total medical expenditure are allocated to dialysis patients, which consist of 0.3% of the population, almost at public expense. Of note, although Japan has a universal health insurance system, a third party cannot browse the insurance system to find out about individual treatment history. The medical costs as medical information within a single facility can be examined. Therefore, calculation of the medical or drug costs when visiting other hospitals, transport costs, and labor costs is difficult. Although the Japanese National Health Insurance database (Kokuho database [KDB]) exists, it does not store data on renal function^19^.

There are many problems associated with dialysis care such as too many cases of emergent dialysis that need to use a central venous catheter (CVC), life-prolonging dialysis, and early death after initiation of dialysis therapy. In particular, some studies report that the unplanned initiation of dialysis using a CVC is associated with mortality^20–22^. As this emergent initiation of dialysis increases mortality and costs, the challenge is to find ways of avoiding its use. The present study examines the relationship between the condition of the patient at their first visit to the nephrologist and at the initiation of dialysis therapy and the costs of dialysis initiation and prognosis as there are no such reports in Japan.

## Results

### Characteristics of enrolled patients

Backgrounds of enrolled patients are shown in Table 1A. About 45 female patients (29.8%) were included. The average age at the time of dialysis initiation was 65.7 ± 14.7 years and 25 patients (16.6%) aged ≥ 80 years. As the primary cause of ESRD, 45 (29.8%), 38 (25.2%), and 35 (23.2%) patients had diabetes mellitus (DM), chronic glomerulonephritis (CGN), and nephrosclerosis, respectively. As for past histories and complications, 57 (37.7%), 133 (88.1%), 63 (41.7%), 21 (13.9%), and 72 (47.7%) patients had DM, hypertension, heart disease, stroke, and cardiovascular disease (CVD), respectively. Overall, 32 patients (21.1%) had not been consulted by nephrologists for over 6 months. Emergent initiation of dialysis was conducted for 71 patients (47%), and 80 patients (53%) had planned initiation of dialysis. The reasons for the emergent initiation of dialysis were heart failure (n = 20), uremia (n = 18), AKI or RPGN (n = 11), hyperkalemia and/or acidosis (n = 3), and others (n = 19). Others included cases that initiated emergent dialysis in a comprehensive judgment, despite the absence of a pressing reason. Patient data at the time of dialysis initiation are shown in Table 1B. The average serum albumin was 3.13 ± 0.6 g/dL. The average concentration of C-reactive protein (CRP) was 1.46 ± 3.08 mg/dL. The average medical costs to the hospital were 6.08 ± 3.86 × 10^4^ JPY/day because they were the total costs during hospitalization; however, if treating the systemic disease required procedures other than dialysis, the costs of those were also included. Backgrounds of enrolled patients and patient data at the time of dialysis initiation of emergent and planned groups are shown in Table 2A and B respectively.

**Table 1:** (A) Patient backgrounds, (B) Patient data at the dialysis initiation.

			Total (n = 151)			Death ≤ 2 Y (n = 26)			Alive (n = 125)		
**(A)**											
**Age (year; mean ± SD)**			65.7 ± 14.7			75.2 ± 13.4			63.8 ± 14.3		
≥ 80 (% [n])			16.6 (25)			46.2 (12)			10.4 (13)		
Gender ; male (% [n])			70.2 (106)			65.4 (17)			71.2 (89)		
**Primary cause of ESRD**											
DM (% [n])			29.8 (45)			30.8 (8)			29.6 (37)		
CGN (% [n])			25.2 (38)			19.2 (5)			26.4 (33)		
Nephrosclerosis (% [n])			23.2 (35)			7.7 (2)			26.4 (33)		
Others (% [n])			21.8 (33)			42.3 (11)			17.6 (22)		
**Past history and complications**											
DM (% [n])			37.7 (57)			34.6 (9)			38.4 (48)		
Hypertension (% [n])			88.1 (133)			69.2 (18)			92.0 (115)		
Heart disease (% [n])			41.7 (63)			61.5 (16)			37.6 (47)		
Stroke (% [n])			13.9 (21)			30.8 (8)			10.4 (13)		
CVD (% [n])			47.7 (72)			76.9 (20)			41.6 (52)		
Cancer (% [n])			17.9 (27)			30.8 (8)			15.2 (19)		
AKI (% [n])			13.2 (20)			61.5 (10)			8.0 (10)		
Nephrologist’s care < 6 months (% [n])			21.1 (32)			53.8 (14)			14.4 (18)		
**CKD stage at first visit**											
1 (% [n])			3.3 (5)			0 (0)			4.0 (5)		
2 (% [n])			9.9 (15)			7.7 (2)			10.4 (13)		
3 (% [n])			17.2 (26)			11.4 (3)			16.8 (21)		
4 (% [n])			31.1 (47)			34.6 (9)			30.4 (38)		
5 (% [n])			39.7 (60)			46.2 (12)			38.4 (48)		
Emergent initiation (% [n])			47.0 (71)			92.3 (24)			37.6 (47)		
Peritoneal dialysis (% [n])			10.6 (16)			3.8 (1)			12.0 (15)		
**(B)**											
Body surface area (m^2^; mean ± SD)			1.65 ± 0.20			1.57 ± 0.16			1.66 ± 0.20		
Systolic blood pressure (mmHg; mean ± SD)			151.8 ± 24.4			139.3 ± 28.3			154.3 ± 22.8		
CTR in chest X-ray (%; mean ± SD)			52.6 ± 6.7			58.0 ± 7.9			51.5 ± 5.8		
EF in ultrasonic cardiography (%; mean ± SD)			64.2 ± 12.2			58.4 ± 18.1			65.2 ± 10.7		
eGFR (ml/min/1.73 m^2^; mean ± SD)			6.16 ± 3.89			9.80 ± 1.40			5.40 ± 2.16		
Hb (g/dl; mean ± SD)			9.21 ± 1.31			9.16 ± 1.67			9.22 ± 1.23		
Alb (g/dl; mean ± SD)			3.13 ± 0.60			2.69 ± 0.73			3.22 ± 0.52		
CRP (mg/dl; mean ± SD)			1.46 ± 3.08			4.93 ± 5.60			0.74 ± 1.44		
Medical costs (× 10^4^ JPY; mean ± SD)			257 ± 316			611 ± 564			183 ± 159		
Period of hospitalization (days; mean ± SD)			42.9 ± 45.3			79.7 ± 87.2			35.2 ± 26.1		
Medical costs per day (× 10^4^ JPY/day; mean ± SD)			6.08 ± 3.86			10.0 ± 7.97			5.27 ± 1.15		

Data are presented as mean ± standard deviation (range) unless otherwise stated.

*ESRD* end-stage renal disease, *DM* diabetes mellitus, *CGN* chronic glomerular nephropathy, *CVD* cardiovascular disease, *AKI* acute kidney injury, *CKD* chronic kidney disease, *CTR* cardio thoracic ratio, *EF* ejection fraction, *JPY* Japanese Yen.

**Table 2:** (A) Patient backgrounds of planned and emergent groups, (B) patient data at the dialysis initiation of Planned and Emergent groups.

	Total (n = 151)	Planned (n = 80)	Emergent (n = 71)
**(A)**			
Age (year; mean ± SD)	65.7 ± 14.7	64.1 ± 13.9	67.6 ± 15.5
≥ 80 (% [n])	16.6 (25)	10 (8)	23.9 (17)
Gender; male (% [n])	70.2 (106)	66.3 (53)	70.2 (53)
**Primary cause of ESRD**			
DM (% [n])	29.8 (45)	21.3 (17)	39.4 (28)
CGN (% [n])	25.2 (38)	32.5 (26)	16.9 (12)
Nephrosclerosis (% [n])	23.2 (35)	26.3 (21)	19.7 (14)
Others (% [n])	21.8 (33)	20 (16)	23.9 (17)
**Past history and complications**			
DM (% [n])	37.7 (57)	31.3 (25)	45.1 (32)
Hypertension (% [n])	88.1 (133)	95 (76)	80.3 (57)
Heart disease (% [n])	41.7 (63)	31.3 (25)	53.5 (38)
Stroke (% [n])	13.9 (21)	5 (4)	23.9 (17)
CVD (% [n])	47.7 (72)	33.8 (27)	63.4 (45)
Cancer (% [n])	17.9 (27)	15 (12)	21.1 (15)
AKI (% [n])	13.2 (20)	1.3 (1)	26.8 (19)
Nephrologist’s care < 6 months (% [n])	21.1 (32)	8.8 (7)	35.2 (25)
**CKD stage at first visit**			
1 (% [n])	3.3 (5)	6.3 (5)	0 (0)
2 (% [n])	9.9 (15)	12.5 (10)	7.0 (5)
3 (% [n])	17.2 (26)	18.8 (15)	12.7 (9)
4 (% [n])	31.1 (47)	26.3 (21)	36.6 (26)
5 (% [n])	39.7 (60)	36.3 (29)	43.7 (31)
Peritoneal dialysis (% [n])	47.0 (71)	16.3 (13)	4.3 (3)
**(B)**			
Body surface area (m^2^; mean ± SD)	1.65 ± 0.20	1.63 ± 0.18	1.68 ± 0.21
Systolic blood pressure (mmHg; mean ± SD)	151.8 ± 24.4	152.4 ± 22.9	151 ± 26.1
CTR in chest X-ray (%; mean ± SD)	52.6 ± 6.7	50.5 ± 6.2	54.9 ± 6.5
EF in ultrasonic cardiography (%; mean ± SD)	64.2 ± 12.2	66.0 ± 9.5	62.2 ± 14.5
eGFR (ml/min/1.73 m^2^; mean ± SD)	6.16 ± 3.89	5.29 ± 1.75	7.14 ± 5.21
Hb (g/dl; mean ± SD)	9.21 ± 1.31	9.55 ± 1.09	8.82 ± 1.44
Alb (g/dl; mean ± SD)	3.13 ± 0.60	3.32 ± 0.51	2.91 ± 0.61
CRP (mg/dl; mean ± SD)	1.46 ± 3.08	0.67 ± 1.46	2.35 ± 4.05
Medical costs (× 10^4^ JPY; mean ± SD)	257 ± 316	149 ± 100	379 ± 418
Period of hospitalization (days; mean ± SD)	42.9 ± 45.3	30.0 ± 17.5	57.4 ± 60.5
Medical costs per day (× 10^4^ JPY/day; mean ± SD)	6.08 ± 3.86	5.15 ± 1.21	7.14 ± 5.30

Data are presented as mean ± standard deviation (range) unless otherwise stated.

*ESRD* end-stage renal disease, *DM* diabetes mellitus, *CGN* chronic glomerular nephropathy, *CVD* cardiovascular disease, *AKI* acute kidney injury, *CKD* chronic kidney disease, *CTR* cardio thoracic ratio, *EF* ejection fraction, *JPY* Japanese Yen.

### Overall survival and associated factors

**Figure 1:**
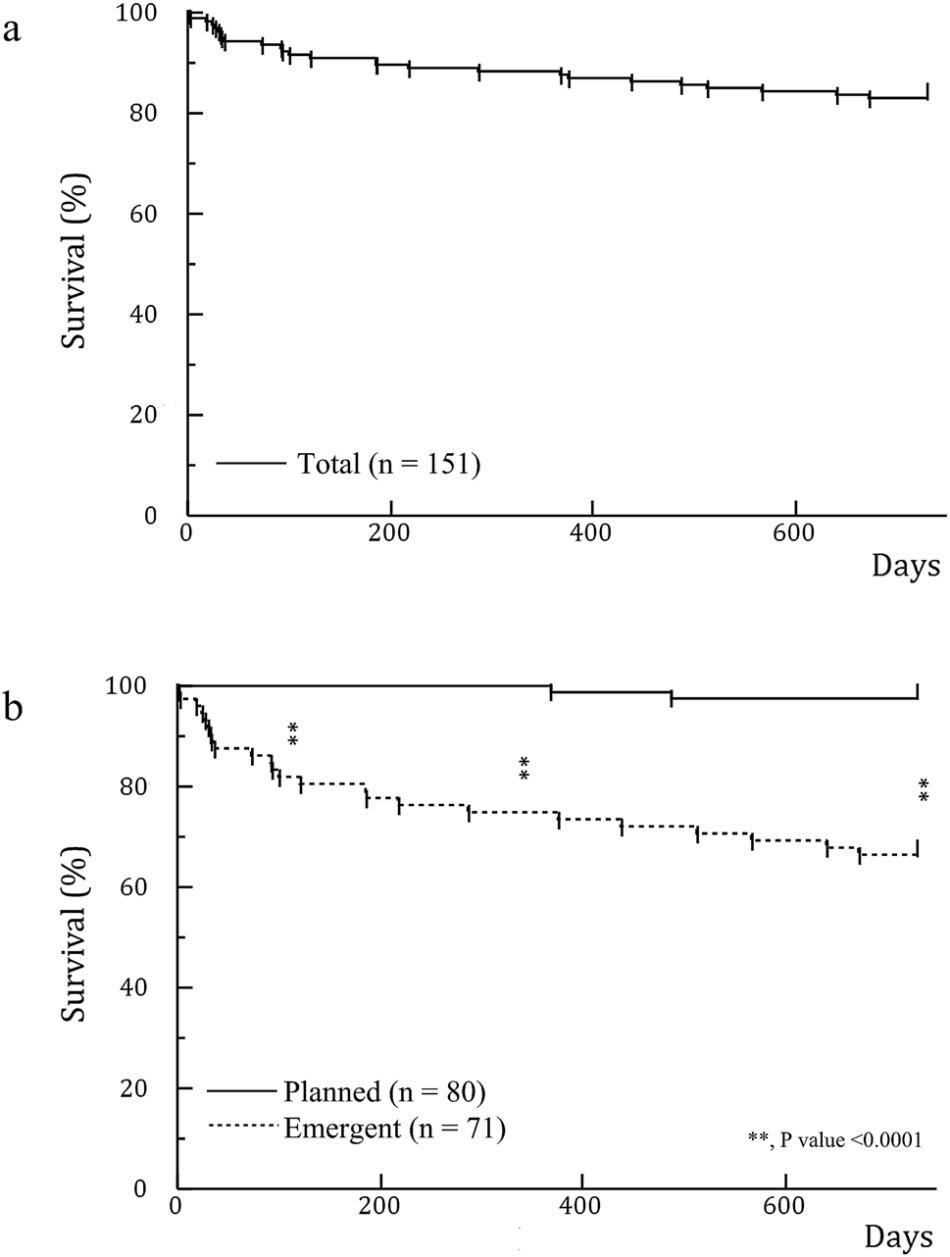
**(a)** Survival rate in all patients. The survival rate after initiation of dialysis was 91.4% in 4 months and 82.8% in 2 years. **(b)** Survival rate comparing emergent and planned initiation. Emergent initiation led to a significantly higher mortality rate than planned initiation of dialysis (p value < 0.0001) in each of 4 months, 1 year, and 2 years in the log-rank test. All patients who died within 4 months had undergone emergent initiation of dialysis.

The survival rates after initiation of dialysis were 91.4% at 4 months and 82.8% at 2 years (Fig. 1a). Log-rank test revealed that dialysis patients with emergent initiation had significantly higher mortality at each time point than those with planned initiation (Fig. 1b). In particular, all patients who died within 4 months had undergone urgently initiated dialysis.

The factors associated with death within 2 years and each hazard ratio (HR) are shown in Table 3. Univariate analysis showed that high medical costs, emergent initiation, high CRP concentration, old age, low systolic blood pressure before first dialysis, and presence of AKI or CVD were the risk factors of death within 2 years. Multivariate analysis showed that emergent initiation of dialysis led to a 7.00-fold higher risk of death within 2 years compared to planned initiation (HR = 7.00: 95% confidence interval [CI] = 1.78–46.60; P = 0.004). The same multivariate analysis also showed that the risk of death within 2 years increased as the medical costs per day increased by thousand yen (HR = 1.03; 95% CI = 1.02–1.04; P < 0.001) and with increasing CRP concentration (per 0.1 mg/dL; HR = 1.01; 95% CI = 1.00–1.02; P = 0.01).

**Table 3:** Contribution factors to death in 2 years.

	Univariate analysis			Multivariate analysis		
	HR	95% CI	P value	HR	95% CI	P value
Costs (per × 10^3^ JPY/day increase)	1.04	1.03–1.05	< 0.001*	1.03	1.02–1.04	< 0.001*
Emergent initiation (vs. planned)	16.39	4.9–102.0	< 0.001*	7.00	1.78–46.60	0.004*
CRP (per 0.1 mg/dL increase)	1.02	1.02–1.03	< 0.001*	1.01	1.00–1.02	0.01*
BP < 130 mmHg (vs. ≥ 130)	3.57	1.46–7.95	0.007*	2.77	0.91–7.54	0.07
Age ≥ 80 years old (vs. < 80)	4.91	2.23–10.65	< 0.001*	2.13	0.82–5.48	0.12
CVD (vs. without CVD)	4.05	1.72–11.09	0.001*	1.63	0.50–5.83	0.42
Female (vs. male)	1.28	0.54–2.80	0.56	1.43	0.56–3.53	0.44
DM (vs. without DM)	0.86	0.37–1.90	0.72	0.87	0.30–2.47	0.80
AKI (vs. without AKI)	6.02	2.63–13.15	< 0.001*	1.04	0.31–3.00	0.95

*HR* hazard ratio, *CI* confidence interval, *BP* blood pressure, *CVD* cardiovascular disease, *DM* diabetes mellitus, *AKI* acute kidney injury, *JPY* Japanese Yen.

*p value < 0.05.

### Factors associated with increasing medical costs

The factors associated with increasing medical costs and each regression coefficient (β) are shown in Table 4. In the multivariate analysis, increasing CRP (β = 0.003; 95% CI = 0.001–0.005; P = 0.003) and emergent initiation (β = 0.080; 95% CI = 0.021–0.139; P = 0.008) were associated with increasing medical costs per day in hospital for the initiation of dialysis.

**Table 4:** Contributing factors to increasing medical costs.

	Univariate analysis			Multivariate analysis		
	β	95% CI	P value	β	95% CI	P value
CRP (per 0.1 mg/dl increase)	0.004	0.003–0.006	< 0.001*	0.003	0.001–0.005	0.003*
Emergent initiation (vs. planned)	0.115	0.061–0.169	< 0.001*	0.080	0.021–0.139	0.008*
BP < 130 mmHg (vs. ≥ 130)	0.080	− 0.003–0.164	0.06	0.049	− 0.031–0.129	0.23
ALB (per 0.1 g/dl increase)	− 0.016	− 0.025 to − 0.007	< 0.001*	− 0.006	− 0.017–0.004	0.23
CVD (vs. without CVD)	0.031	− 0.026–0.087	0.29	− 0.017	− 0.075–0.041	0.56
Age ≥ 80 years old (vs. < 80)	0.027	− 0.050–0.103	0.49	− 0.020	− 0.095–0.055	0.60
Female (vs. male)	− 0.003	− 0.065–0.059	0.92	0.006	− 0.052–0.064	0.84
DM (vs. without DM)	− 0.009	− 0.068–0.050	0.76	0.006	− 0.061–0.054	0.91

*β* standardized partial regression coefficient, *CI* confidence interval, *BP* blood pressure, *CVD* cardiovascular disease, *DM* diabetes mellitus.

*p value < 0.05.

### Risk factors associated with emergent initiation of dialysis

The factors associated with emergent initiation of dialysis and each odds ratio (OR) are shown in Table 5. In the multivariate analysis, AKI (OR = 25.95; 95% CI = 4.26–508.8; P < 0.001), stroke (OR = 7.04; 95% CI 1.92–31.21; P = 0.003), DM (OR = 2.65; 95% CI = 1.14–6.32; P = 0.02), heart failure (OR = 2.97; 95% CI = 1.03–9.14; P = 0.04), and short-term care from a nephrologist (< 6 months; OR = 3.22; 95% CI = 1.06–10.54; P = 0.04) were associated with the emergent initiation of dialysis.

**Table 5:** Association with emergent initiation.

	Univariate analysis			Multivariate analysis		
	OR	95% CI	P value	OR	95% CI	P value
AKI (vs. no AKI)	28.87	5.72–526.2	< 0.001*	25.95	4.26–508.8	< 0.001*
Stroke (vs. no stroke)	5.98	2.08–21.68	0.001*	7.04	1.92–31.21	0.003*
DM (vs. no DM)	1.81	0.93–3.53	0.08	2.65	1.14–6.32	0.02*
Nephrologist’s care < 6 months (vs. ≥ 6 months)	5.67	2.37–15.17	< 0.001*	3.22	1.06–10.54	0.04*
Heart failure (vs. no heart failure)	5.67	2.37–15.17	< 0.001*	2.97	1.03–9.14	0.04*
Female (vs. male)	0.67	0.32–1.35	0.26	0.49	0.18–1.25	0.14
Age ≥ 80 years old (vs. < 80)	2.83	1.17–7.40	0.02*	2.18	0.66–7.39	0.20
Hypertension (vs. no hypertension)	0.21	0.06–0.64	0.005*	0.37	0.07–1.73	0.20
Ischemic heart disease (vs. no ischemic heart disease)	2.17	1.05–4.59	0.04*	1.17	0.45–2.98	0.74

*OR* odds ratio, *CI* confidence interval, *AKI* acute kidney disease.

*p value < 0.05.

## Discussion

This is the first report from Japan that describes the relationship between the patient status before initiation of dialysis treatment, medical costs, and prognosis. Furthermore, it was our understanding that the backgrounds of the enrolled patients were not particularly different from that of patients in other countries according to previous reports on the subject^10,21,23,24^.

It has been reported that the long-term survival rate of dialysis patients in Japan is excellent compared to that in other countries^25^. However, some reports have shown that the survival rate within 4 months of the dialysis initiation was not much different from that of other countries^25–28^. In this study, the survival rate within 2 years was about the same as that stated in the latest JSDT Renal Data Registry^17^, and the rate of early death (within 4 months after the initiation of dialysis) was the same as that in Japan and other countries.

Rayner et al. reported that 15–50% of patients undergoing dialysis were initiated using temporary catheters^29^. Some reports have described that emergent initiation using a short-term CVC is associated with high rates of mortality and hospitalization^20–22,30^. In this study, 47% of all patients experienced emergent initiation, which was not much different from that found in pre-existing studies. We showed that in each time point after 4 months, 1 and 2 years, emergent initiation affected a higher mortality than planned initiation of dialysis (Fig. 1b). Furthermore, in our multivariate analysis, emergent initiation affected an increase in mortality at 2 years and medical costs for dialysis initiation. Jeong et al. reported that, although the initiation of dialysis using a CVC was associated with a worse rate of survival, CVC-related complications including infection and the duration of CVC use did not affect survival^23^. Therefore, considering that patients who have experienced emergent initiation of dialysis required rapid care, it could be estimated that emergent initiation and poor systemic state at that time worsened the survival rate for patients rather than CVC use itself.

Bazeley and Ishii reported that CRP was positively associated with mortality^31,32^. Indeed, the present study showed that factors affecting the increase in medical costs were not only emergent initiation but also high CRP levels.

Other factors contributing to death in dialysis patients have been examined in many studies. Previous studies revealed that late referral (the duration from first visit to nephrologists to initiation of dialysis was < 3 months) was found in 35% of the patients and was associated with increased mortality^33,34^. In this study, we showed that short-term care by nephrologists (< 6 months) was one of the contributing factors to the requirement for emergent initiation of dialysis. Kuragano et al. reported that there was a high risk of death in patients with hemoglobin levels outside the target range of 10–11 g/dL^35^, whereas other reports showed that low levels of serum albumin were associated with an increased mortality risk in dialysis patients^36,37^. In this study, neither anemia nor serum levels of albumin had an impact on mortality. According to a report by Kurella et al., despite an increase in the number of elderly patients, the 1-year survival rate of elderly patients has not changed compared to 1996 and 2002^38^. Lamping et al. reported that although more complications, the worse the prognosis, and aging alone did not affect the prognosis^39^. Indeed, we also did not find that elderly patients had poor prognosis, although increase in elderly patients is a characteristic of Japanese dialysis medicine and that the average age of dialysis initiation increases year by year and exceeds 69 years; approximately 33% of all patients are ≥ 75 years^17^.

A report by Takura et al. was one of the few studies to verify the cost-effectiveness of dialysis treatment in Japan^18^. However, the sample appeared to be limited possibly due to the difficulty of accessing Japanese insurance data or of following-up. In the present study, we found that the medical costs of hospitalization at the time of initiation of dialysis are associated with the prognosis, although we only examined the simple cost of hospitalization. The results of increased costs due to unscheduled AVF creation from Thamer et al. were similar to our findings^40^.

As we found that emergent initiation of dialysis increases both mortality and medical costs, we also examined the risk factors leading to emergent initiation and found that AKI, DM, heart failure, and short-term care (< 6 months) by nephrologists are the risk factors. Thus, it was revealed that a good long-term survival rate could not be obtained if dialysis was initiated emergently at high medical costs. Although present findings were analyzed in Japan, avoiding emergent initiation may universally lead to better prognoses and medical costs for patients with ESRD.

There were some limitations to this study. First, although high CRP levels were a factor that worsened the prognosis for dialysis patients along with emergent initiation in this study, measurement of CRP levels remains uncommon in North America. In addition, the median baseline CRP level in Japan (1.0 mg/L) is much lower than that in other countries (6.0 mg/L)^31^. Because we did not examine the relationship between CRP, a non-specific inflammatory marker, and the patient’s background, the reasons behind the levels of CRP found were unclear. Second, although it was confirmed in an existing study^23^, this study did not show whether CVC-related complications or the term of CVC use affected prognosis. Third, because this was a retrospective observational study, further long-term observational or prospective studies are needed to confirm our findings.

In conclusion, for Japanese ESRD patients, emergent dialysis initiation was a contribution factor to both death and increasing medical costs for the initiation of dialysis. To avoid emergent initiation, those patients should be referred to nephrologists early. Planned initiation of dialysis should be undertaken when treating ESRD patients with histories of AKI, stroke, DM, and heart failure.

## Methods

We conducted a retrospective observational study named “Observational Study for the Optimal Initiation of Dialysis treatment”. Present paper 'Emergent initiation of dialysis is related to an increase in both mortality and medical costs' was described based on some of the results of this study. All procedures performed in this study involving human participants were in accordance with the ethical standards of the institutional and/or national research committee and the 1964 Helsinki declaration and its later amendments or comparable ethical standards. The study protocol was approved by the Ethics Committee of Juntendo University Hospital, Tokyo, Japan (approval number 17-122). Informed consent was obtained in a manner approved by the Ethics Committee from all individual participants included in this study. This study is registered in the clinical trials registry (No. UMIN000037871).

The primary endpoint of this study was the 2-year survival rate of patients following the initiation of dialysis and the secondary endpoints were the medical costs and the number of days in hospital required for the initiation of dialysis.

### Patients

The inclusion criterion for this study was the initiation of dialysis therapy, both HD and PD, from September 2013 to August 2015 at the Juntendo University Hospital, Tokyo, Japan (n = 176 patients). The exclusion criteria were kidney transplantations within 2 years after the initiation of dialysis (n = 2 patients), refusal to participate in this study (n = 1 patient), and loss to follow-up (n = 22 patients). In total, 151 Japanese patients with ESRD were enrolled in this study.

### Initiation

Patients were divided into two groups according to whether dialysis was initiated in a planned or emergent manner. Planned initiation was defined as the cases in which patients had been given therapeutic interventions previously including hypertension control, early therapy for anemia and mineral disorders, and diet recommendations by nephrologists before starting dialysis therapy. Their permanent vascular access (VA) was placed before the initiation of dialysis therapy in every case. On the other hand, emergent initiation was defined as the cases in which HD patients whose permanent VA had not been placed and PD patients whose peritoneal catheter had not been placed before initiation of dialysis therapy were included, regardless of whether or not they had been seen by nephrologists. Patients who started unplanned HD therapy used a short-term CVC in every case, because they either could not wait for the placement of catheters or the maturation of VA.

### Definition of etiology for ESRD

Although there are patients with DM as both complication and primary cause of ESRD, when we consider the associated factor in this study, we referred to a complication of DM. Hypertension involves cases diagnosed previously or with a history of medication. Heart disease includes heart failure, ischemic heart disease, arrhythmia, and valvular heart disease. Stroke includes cerebral infarction, cerebral hemorrhage, and subarachnoid hemorrhage. Using the guideline for management of CVD^41^, heart disease, stroke, and peripheral artery disease were defined as CVD.

### Medical costs and period of hospitalization

When we considered the medical costs and period of hospitalization for preparing and initiating dialysis therapy, hospitalization for the creation of VA or for the stepwise initiation of PD using the Moncrief and Popovich technique were included in this study even without initiate of dialysis.

### Follow-up

We contacted the satellite dialysis clinics to obtain clinical information of the patients after the discharge from our hospital including date of death, cause of death, or date of kidney transplantation.

### Statistical analysis

The clinical characteristics of patients were summarized as mean ± standard deviation for continuous variables and counts and proportions for categorical variables. In this study, the relationship between emergent dialysis initiation and costs or the prognostic factors were investigated. Overall survival rate was estimated by the Kaplan–Meier method. The difference in survival rates between the two groups was examined using a log-rank test. The univariate and multivariate Cox proportional hazard model was conducted to evaluate the impact of emergent dialysis on survival and the HR, or the 95% CI was calculated. Second, the relationship between medical costs and emergent dialysis initiation was assessed by simple and multiple regression analyses with medical costs as the dependent variable. Finally, to identify the risk factors for emergent dialysis initiation, univariate and multivariate logistic regression were performed. Clinical variables for univariate and multivariate analyses other than emergent dialysis initiation were selected based on prior knowledge about the variables and all selected variables were included in the multivariate analysis, regardless of the results of the univariate analysis. All statistical analyses were performed using the Windows version of JMP 12 (SAS Institute Inc., Cary, NC, USA), and p values of < 0.05 were considered statistically significant.
